# ‘Early Life Adversity and Social Cognition in the General Adult Population: A Systematic Review and Meta-Analysis’

**DOI:** 10.1007/s40653-025-00724-y

**Published:** 2025-06-24

**Authors:** Michael Mackey, Ellen Dunne, Elayne Ahern

**Affiliations:** 1 Department of Psychology, University of Limerick, Castletroy, Limerick, V94 T9PX Ireland; 2 School of Psychology, University College Dublin, Belfield, Dublin 4, Ireland; 3 Health Research Institute, University of Limerick, Castletroy, Limerick, V94 T9PX Ireland

**Keywords:** Early life adversity, Trauma, General population, Social cognition, Emotion recognition and theory of mind

## Abstract

Early life adversity (ELA) is associated with diminished social cognition in populations with a neuropsychiatric diagnosis however less is known about general populations. Examining the association between ELA and social cognition in non-clinical populations allows determinants of social cognition to be explored in the absence of psychiatric classifications. The aim of this study was to examine the association between ELA and social cognition in the general adult population. The protocol for this study was preregistered on Prospero (preregistration-ID: CRD42023433358). Four databases and reverse citation searches were performed to identify relevant articles examining the relationship between social cognitive domains and ELA. Fisher’s r-to-z transformed correlation coefficients were synthesised using inverse variance weighted random effects modelling to provide a mean effect size estimate, with 95% confidence intervals. The limited number of included studies precluded subgroup, sensitivity and publication bias analysis. 1,314 articles were identified with 20 articles representing 18 study populations meeting the inclusion criteria. There is preliminary evidence that ELA is associated with diminished theory of mind (*z*_*r*_ = -.247, *p* = .002) and emotion recognition (*z*_*r*_ = -.121, *p* < .001) in the general adult population. Sexual abuse as a subdomain of ELA is also associated with diminished emotion recognition (*z*_*r*_ = -.056, *p* < .001). Correlational findings on the association between retrospective accounts of ELA and social cognitive deficits during adulthood suggest a continuing impact of trauma in later life. Longitudinal research is best situated to further explore causative and mediating factors underlying this relationship. These results have helped to clarify the ELA-social cognition association without potential confounds of concurrent mental health status, and, further elucidate a possible risk mechanism for functional disability.

**Supplementary Information:**

The online version contains supplementary material available at 10.1007/s40653-025-00724-y.

## Introduction

Early life adversity (ELA) describes exposure to severe adverse experiences that deviate from an expected environment prior to reaching eighteen years of age, necessitating significant psychological and biological adaptation (McLaughlin, 2016). These experiences have been encapsulated through various operational definitions including adverse childhood experiences (ACE), childhood abuse (physical, emotional and sexual), childhood neglect (physical and emotional) and childhood trauma. Estimates from the World Health Organisation suggest that 39% of the global population have been exposed to early life adversity (Kessler et al., 2010). In addition to increasing the risk of being diagnosed with a neuropsychiatric disorder throughout life (Clark et al., 2010; Lowthian et al., 2021), the financial burden associated with ELA exposure is significant and is estimated to range between 1–6% of European countries Gross Domestic Product (GDP) annually (Hughes et al., 2021). Research has indicated that individuals exposed to ELA are more likely to use avoidant emotion focused coping strategies during times of distress and are more likely to engage in health harming behaviours such as smoking and harmful alcohol use (Hughes et al., 2021; Sheffler et al., 2019). Given the significant personal, interpersonal and wider societal burden associated with ELA, understanding how components of cognitive wellbeing including social cognition are impacted is pertinent.

Social cognition describes the process of perceiving, interpreting and using information relevant to our social world (Baron et al., 1998). Social cognition research has centred around studying mental representations of oneself, others and the physical world in which we live, typically encompassing aspects of theory of mind, emotion recognition, and metacognitive processes (Augoustinos et al., 2014). Theory of Mind is a subdomain of social cognition that describes the ability to represent human mental states and make inferences about other people’s intentions, beliefs and emotions (Baron et al., 1998; Baron-Cohen et al., 2001). Emotion recognition describes the ability to identify other people’s feelings and is thought to reflect lower-level perceptual processing of emotional information, while theory of mind describes the processing of cognitive information reflecting higher level integration (Barbato et al., 2015; Mitchell & Phillips, 2015).

Alexithymia is characterised by difficulties experiencing, identifying and expressing emotions, that has been succinctly described as having “no words for feelings” (Samur et al., 2013; Taylor et al., 1997). Alexithymia is associated with deficits in emotion recognition (Grynberg et al., 2012). Metacognitive processes have also been identified as central for validating schema representations by looking for environmental and interpersonal appraisals (Schwarz et al., 2007).

Social cognition has been studied extensively in individuals diagnosed with neuropsychiatric disorders. Evidence synthesis articles have noted social cognitive deficits in populations diagnosed with major depressive disorder (Bora & Berk, 2016), schizophrenia (Sprong et al., 2007), bipolar disorder (Samamé et al., 2012), post-traumatic stress disorder (Stevens & Jovanovic, 2019) and anxiety disorders (Plana et al., 2014) when compared with controls. The aggregation of this research has resulted in theory of mind deficits being proposed as a trait marker for schizophrenia spectrum disorder and bipolar disorder (Bora et al., 2009) and a transdiagnostic marker of neurological differences associated with neuropsychiatric disorders (Cotter et al., 2018).

Attachment science has provided a framework to understand how ELA exposure may influence mental representations of oneself and others (Bowlby, 1969). Of relevance to social cognition is the ‘defensive exclusion’ hypothesis that proposes that insecurely attached individuals filter out relevant information related to an attachment figure if causing emotional pain (Bowlby, 1980). This selective processing of information is thought to influence theory of mind capabilities through biased processing of social information (Leppänen & Nelson, 2009; Pollak, 2008; Vanwoerden et al., 2015). ELA has been described as the most destructive factor for the development of attachment styles and is associated with attachment insecurities in adulthood (Erozkan, 2016; Fonagy, 2018; Styron & Janoff-Bulman, 1997). Within interpersonal relationships, higher levels of ELA were associated with negative self-perception during recent social interactions, reduced social motivation, a lower sense of belonginess and higher desire for social avoidance (Steenkamp et al., 2023).

The current literature examining the relationship between ELA and social cognition is sparse, with the vast majority focused on neuropsychiatric populations. There is evidence that ELA is trans-diagnostically associated with psychopathology risk and reactivity to stress in later life (Albott et al., 2018). One systematic review uncovered that ELA exposure is trans-diagnostically associated with social cognition deficits in clinical populations diagnosed with schizophrenia, bipolar disorder, borderline personality disorder and posttraumatic stress disorder (Rokita et al., 2018). A recent meta-analysis did not find a significant association between ELA and social cognition in people diagnosed with psychotic disorders suggesting that the transdiagnostic association is not consistently observed across populations while acknowledging the need for further research in this area to substantiate these findings (Fares-Otero et al., 2023).

Understanding putative causative and mediating factors of social cognitive deficits is of crucial importance due to its association with individual functioning and wellbeing. A meta-analysis uncovered that theory of mind is strongly associated with community functioning which includes independent living skills, social and work functioning in a population of individuals diagnosed with schizophrenia (Fett et al., 2011). In addition, theory of mind is significantly associated with quality of life scores in people diagnosed with schizophrenia (Maat et al., 2012). Furthermore, there is evidence that social cognition skills are modifiable. A meta-analysis has provided evidence that socio-cognitive training can increase scores in social cognition tasks in both children and adults in a healthy population study however, transfer effects are yet to be determined (Roheger et al., 2022).

In summary, there is distinct evidence that ELA is associated with significant behavioural, emotional, neurobiological and psychological adaptation that can influence social cognition and adult health outcomes (Kuzminskaite et al., 2021; Nelson et al., 2020; Steenkamp et al., 2023). The breadth of evidence examining the relationship between ELA and social cognition is sparse and centred on populations with a neuropsychiatric diagnosis. Novel to this review article is the exploration of an association between ELA and social cognition in the general population which allows for the determinants of social cognition to be explored in the absence of psychiatric classifications. This will assist in discerning if negative associations detected in populations with a neuropsychiatric diagnosis persist in non-clinical populations while minimising the impact of limitations associated with neuropsychiatric groupings (Alameda et al., 2023; Rokita et al., 2018). Specifically, we sought to examine subdomains of social cognition including theory of mind and emotion recognition to understand how domains of social cognition may be differentially associated with ELA exposure.

## Methods

This review article followed the Preferred Reporting for Systematic Reviews and Meta-analyses guidelines (PRISMA) with the checklist available in Supplementary Table 1 (Page et al., 2021). The study protocol was preregistered on Prospero (preregistration-ID: CRD42023433358). Amendments to the preregistered protocol are detailed in Supplement 1.

### Search Strategy

A defined search of PsycInfo (https://www.apa.adaorg/pubs/databases/psycinfo), PubMed (www. pubmed.ncbi.nlm.nih.gov), Scopus (www.scopus.com) and the Web of Science (www.webofscience.com) was performed to identify potentially relevant records that were published before the 31 st October 2023. A pilot search was initially performed to determine the precision of search terms required to capture potentially relevant papers within each database (PubMed, PsycArticles, Scopus and Web of Science). Pilot systematic searches of key terms within the full article uncovered a substantial volume of irrelevant studies. Pilot searching within the abstract uncovered reduced volumes while retaining potentially relevant studies. Abstract searching of key terms was conducted within the four identified databases. The title/abstract and title/abstract/key search functions were used on the Pubmed and Scopus databases respectively. The search strategy consisted of four key search clusters related to (i) early life adversity, (ii) social cognition, (iii) general population and (iv) association, searched through the use of the ‘AND’ Boolean operator. Pilot searching using association as a key search term within the PsycArticles database resulted in a drastically reduced volume of identified studies. A more conservative search strategy was pursued that did not include association as a key search term for the PsycArticles database to minimise the risk of missing potentially relevant studies. Additional articles were identified through backward citation searches. The exact search strategy for each database can be found in Supplementary Table 2.

### Study Selection and Inclusion/Exclusion Criteria

Studies that consist of human participants aged eighteen years old and older that report quantitative associations between early life adversity and social cognition, peer-reviewed and published in the English language were included. Studies including individuals deemed to be at a higher risk for neuropsychiatric disorder diagnosis were included if no clinical diagnosis was present. Studies including individuals diagnosed with a neuropsychiatric disorder according to the Diagnostic and Statistics Manual (DSM) or International Classification for Disease (ICD) were excluded as well as study populations with chronic health diagnoses including autoimmune disorders, cancer, diabetes, heart disease and neurological disorders (American Psychiatric Association, 2013; World Health Organization, 2019). The full inclusion and exclusion criteria can be found in Supplementary Table 3. The population, exposure, comparator and outcomes (PECO) are listed in Supplementary Table 4.

### Study Selection and Data Extraction

Upon completion of systematic database searches, studies were imported to Covidence (https://www.covidence.org/). Two independent reviewers (R1 & R2) screened the articles for inclusion at the title/abstract level. The Covidence software was used to blind each reviewer to the decisions being made by the other reviewer. Disagreements occurred when one reviewer chose to include an article and the other reviewer chose to exclude the same article. These disagreements were addressed by the two reviewers discussing their rationale. A third reviewer (R3) was consulted when a decision could not be made. The second reviewer provided blind independent assessment for the purposes of inter-rater reliability. An initial pilot of thirty studies was conducted to determine the suitability of the inclusion and exclusion criteria and whether such criteria could be consistently applied within the review pair. A concordance rate of 96.7% was recorded for the thirty-study pilot at the title/abstract level with a Cohen’s kappa of 0.65 reflecting substantial inter-rater reliability (Cohen, 1960).

At the title/abstract level a concordance rate of 88.7% was detected with a Cohens kappa of 0.34. At the full-text level, a concordance rate of 93.1% with a Cohens Kappa of 0.77 was detected reflecting substantial agreement. An exclusion hierarchy (Supplementary Table 5) was used to ensure the rationale for exclusion was consistent at the full-text level. The Covidence software was used for recording decisions.

Data was extracted solely by R1. Second person verification for all extracted material was performed by R2. Any conflicts were resolved through R1 and R2 discussing their rationale. R3 was consulted in the case of any persisting conflicts. Study authors were contacted to obtain any relevant information that was not included in the publication. Extracted data reported in Table 1 included: Article name, citation, sample size, mean age of sample, percentage of each sex within sample, covariate information (age, educational attainment, parental educational attainment, marriage status, ethnicity, mental health indices and socioeconomic status), tests used to measure early life adversity, early life adversity categories, tests used to measure social cognition, social cognition categories, correlation between early life adversity and social cognition score and study design.

**Table 1:** Overview of included studies

First Author	Sample Description	Sample Size (N)	Age in Years (SD)	Sex (% Fem)	Covariates	Adversity Measure	Adversity Type	Social Cognition Measures	Social Cognition Domains Measured	Associations/Correlations	Design
Bérubé et al., (2020)	All mothers with children between the ages of 2–5 years. Participants were recruited from local community organisations that provide services to vulnerable families in Canada	58	Not Stated	100%	No covariates included in correlation of interest	CTQ-SF (French Version)—28 item questionnaire (Bernstein et al., 1994; Paquette et al., 2004)	Physical abuse, physical neglect, emotional abuse, emotional neglect, sexual abuse and aggregate childhood trauma score	Novel method similar to Facial Expression Megamix task (Young et al., 1997)	Emotion recognition—Correctly identifying emotions	CTQ total x Emotion recognition total: (r = −0.29, *p* = 0.03)	Retrospective
Chen et al., (2012)	PennTwins Cohort, a population-based sample of twins born in Pennsylvania, USA between 1959 and 1978. Sample is skewed towards younger unmarried female adults	2752	Age range 20–55	58.5%	Age, SES, Gender and social desirability	CTQ–SF, 28 item questionnaire (Bernstein et al., 1994)	Physical abuse, physical neglect, emotional abuse, emotional neglect, sexual abuse and aggregate childhood trauma score	Social Information Processing-Attribution and Emotional Response Questionnaire (SIP-AEQ; Coccaro et al. 2009) Subscales: Hostile Attribution Biases (HAB), Negative Emotional Responses (NER)	Social Information Processing (SIP) & Negative emotion perception bias	HAB x: CTQ total (r = 0.18, *p* < 0.001), Physical abuse (r = 0.10, *p* < 0.001), Emotional abuse (r = 0.16, *p* < 0.001) NER x: CTQ total (r = 0.10, *p* < 0.001), Physical abuse (r = 0.02, *p* > 0.05), Emotional abuse (r = 0.13, *p* < 0.001)	Retrospective
Dayton et al., (2016)	All pregnant women. The majority of participants were single, either Caucasian or African American and were recruited through fliers at public locations and agencies serving low income families	120	26 (5.7)	100%	None reported	CTQ −28-items (D. Bernstein & Fink, 1998)	Physical abuse, physical neglect, emotional abuse, emotional neglect, sexual abuse and aggregate childhood trauma score	The Infant Facial Expressions of Emotion from Looking At Pictures (IFEEL) task—30 pictures (Emde et al.,1993)	Emotion recognition – Negative emotion perception bias	IFEEL total x CTQ subdomains: EA: (r = 0.12, *p* > 0.05), PA: (r = −0.02, *p* > 0.05), SA: (r = 0.11, *p* > 0.05), EN: (r = 0.16, *p* > 0.05), PN: (r = 0.05, *p* > 0.05)	Longitudinal design with ELA measured retrospectively
English et al., (2018)	All female undergraduate university students	126	18.98	100%	None reported	CTQ-SF, 25 item questionnaire (Bernstein et al., 2003)	Physical abuse, physical neglect, emotional abuse, emotional neglect, sexual abuse and aggregate childhood trauma score	Images obtained from the Cohn-Kanade Facial Expression database (Kanade, Cohn, & Tian, 2000). A total of 80 target images were presented to each participant identical to those used by Pollak et al. (2009)	Emotion recognition – Amount of information required to accurately identify emotion	CTQ total (low cognitive load condition) x: ER total: (r = −0.06, *p* > 0.05), Fear: (r = −0.09, *p* > 0.05), Anger: (r = −0.05, *p* > 0.05)	Retrospective
Hartling et al., (2019)	Recruited from general population in Germany, representing the middle range of SES and are of European descent	170	43.2 (19.9)	44%	Age, gender and IQ	CTQ (German version)—28 item questionnaire (Bernstein and Fink, 1998)	Physical abuse, physical neglect, emotional abuse, emotional neglect, sexual abuse and aggregate childhood trauma score	Novel facial emotion recognition, developed in alignment with ‘Face Puzzle Explicit’ task (Kliemann et al., 2013)	Facial emotion recognition—Percentage correctly identified	CTQ total x ER total: (β = −0.095, *p* = 0.181)	Retrospective
**iRELATE** Corley et al., (2024)	Recruited via advertisements in local and national media in Ireland	177	36.19 (12.36)	42%	None included in association of interest	CTQ—28 item questionnaire (Bernstein et al., 2003)	Physical abuse, physical neglect, emotional abuse, emotional neglect and sexual abuse	Emotion Recognition Task—Short version from CANTAB (Robbins et al., 1994)	Emotion recognition	Unstandardised regression coefficients ERT Total x physical neglect, B = 0.044, *p* = 0.057	Retrospective
**iRELATE** Rokita et al., (2021)	Recruited via advertisements in local and national media Ireland. All participants were Caucasian	116	35.01 (11.72)	44.8%	Age and years of education	CTQ—25 item questionnaire with rescaled cut‐off scores (Bernstein et al., 2003)	Physical abuse, physical neglect, emotional abuse, emotional neglect and sexual abuse	RMET (Baron-Cohen et al., 2001), The Hinting Task (Corcoran, Mercer, & Frith, 1995), Emotion Recognition Task (ERT; Robbins et al., 1994)	Theory of mind, Emotion recognition	Unstandardised regression coefficients ERT total and neglect (B = − 3.34, t(113) = − 1.98, *p* = 0.049), Physical neglect and the recognition of disgust (B = − 1.28, t(113) = − 1.96, *p* = 0.052)	Retrospective
Kopera et al., (2020)	People presenting to general practitioner (GP) for treatment of an infection, medical device or prophylactic examination	172	39.65 (10.34)	16.9%	None included in correlation of interest	CTQ-SF (Polish version)—28 item questionnaire (Bernstein et al., 1994)	Physical abuse, physical neglect, emotional abuse, emotional neglect, sexual abuse and aggregate childhood trauma score	RMET Polish version—36 different faces (Baron-Cohen et al., 2001)	Theory of mind	CTQ Emotional abuse/neglect x: RMET Negative (r = 0.001, *p* > 0.05), RMET Positive (r = −0.23, *p* < 0.05), RMET Neutral (r = −0.1, *p* > 0.05),	Retrospective
Krammer et al., (2016)	All older adults from the Swiss-German part of Switzerland with a history of child labour or being in foster care	116	77 (7.1)	40.5%	None included in correlation of interest	CTQ (German version)—25 item questionnaire (Bernstein & Fink, 1998)	Physical abuse, physical neglect, emotional abuse, emotional neglect, sexual abuse and aggregate childhood trauma score	Social Acknowledgement Questionnaire (SAQ; Maercker & Müller, 2004), Disclosure of Trauma Questionnaire (DTQ; Müller & Maercker, 2006)	Social Acknowledgement as a trauma victim, Disclosure of Trauma	CTQ total x: Social Acknowledgement (r = −0.30, *p* = 0.001), Dysfunctional disclosure (r = 0.25, *p* < 0.008)	Retrospective
Lee et al., (2019)	Recruited via online notices in three colleges in Daegu, Korea. All participants received financial payment for their participation	201	23.67 (2.76)	59.7%	None included in correlation of interest	CTQ-SF (Korean version) – 28 item questionnaire (Bernstein et al., 2003)	Physical abuse, physical neglect, emotional abuse, emotional neglect, sexual abuse and aggregate childhood trauma score	Difficulties in Emotion Regulation Scale (DERS; Gratz & Roemer, 2004)	Emotional awareness and emotional clarity	CTQ total x: Lack of emotional awareness (r = 0.42, *p* < 0.001), Lack of emotional clarity (r = 0.28, *p* < 0.001)	Retrospective
Liu et al., (2023)	Han Chinese population	134	Median age of 31	68.66%	Age, gender and education	CTQ-SF (Chinese version) – 28 item questionnaire (Bernstein & Fink, 1998; He et al., 2019)	Physical abuse, physical neglect, emotional abuse, emotional neglect, sexual abuse and aggregate childhood trauma score	TAS-20 (Bagby et al., 1994), The Facial Emotion Recognition Test (Gong et al., 2011), The Interpersonal Reactivity Index–C (IRI; Davis, 1983)	Alexithymia, Recognising facial emotions—percentage correctly identified	CTQ total x: TAS-20 total (r = 0.21, *p* = 0.016), TAS subscale DIF (Difficulty identifying feelings; r = 0.219, *p* = 0.012), Recognizing neutral facial emotions (r = − 0.4, *p* < 0.001)	Retrospective
Nweze et al., (2023)	Participants were recruited through the Avon Longitudinal Study of Parents and Children (ALSPAC) in England, United Kingdom	2965	All 24 years old	62.1%	Multivariate regression model controlled the effects of shared variance between the 11 adversity measures	Novel self report questionnaire examining 11 subdomains of childhood adversity reported by parents of children between 0–8 years old	Physical abuse, sexual abuse, inconsistent caregiving, family instability, caregivers abuse, maternal psychopathology, maternal victimization, parental legal problems, parental separation/divorce, financial distress, neighbourhood stress	Emotion Recognition Task (Penton-Voak et al., 2012)	Emotion recognition – Percentage correctly identified	Emotion Recognition total x: Physical abuse (r = 0.02, *p* > 0.05), Sexual abuse (r = −0.06, *p* < 0.01), Inconsistent caregiving (r = 0.07, *p* < 0.001), Family instability (r = 0.01, *p* > 0.05), Caregivers abuse (r = 0.01, *p* > 0.05), Maternal psychopathology (r = 0.00, *p* > 0.05), Maternal victimization (r = −0.02, *p* > 0.05), Parental legal problems (r = −0.03, *p* > 0.05), Parental separation/divorce (r = 0.02, *p* > 0.05), Financial distress (r = −0.03, *p* > 0.05), Neighbourhood stress (r = 0.00, *p* > 0.05)	Longitudinal design with ELA measured retrospectively
Seitz et al., (2022)	Healthy volunteers recruited via advertisements in Germany	35	29.34 (9.69)	80%	None included in correlation of interest	CTQ (German version)—28 item questionnaire (Bernstein et al., 2003; Klinitzke et al., 2012)	Physical abuse, physical neglect, emotional abuse, emotional neglect, sexual abuse and aggregate childhood trauma score	Multiple-choice version of the Movie for the Assessment of Social Cognitionn (MASC; Dziobek et al., 2006)	Theory of mind	MASC total x: CTQ total (r = −0.12, *p* > 0.05)	Retrospective
Terock et al., (2020)	Large general population sample comprising of Caucasians residing in Pomerania, north-eastern Germany (Study of Health in Pomerania; SHIP)	**SHIP‐LEGENDE** 1916 **SHIP-TREND** 3658	**SHIP‐LEGENDE** 55 (13.7) **SHIP-TREND** 51 (15.2)	**SHIP‐LEGENDE** 53% **SHIP-TREND** 51%	Age, sex, education, lifetime diagnosis of MDD	CTQ (German version)—34 item questionnaire (Bernstein & Fink, 1998; Wingenfeld et al., 2010)	Physical abuse, physical neglect, emotional abuse, emotional neglect, sexual abuse and aggregate childhood trauma score	Toronto Alexithymia Scale 20 (TAS-20; Bach et al., 1996, Bagby et al., 1994)	Alexithymia	Unstandardised regression coefficients TAS-20 x: **SHIP-LEGENDE** Neglect (B = 2.88, *p* < 0.001), **SHIP-TREND** Neglect (B = 3.21, *p* < 0.001)	Retrospective
**TestMyBrain** Germine et al., (2015)	Recruited online at TestMyBrain.org, predominantly individuals from the United States (US)	**RMET** 2242 **QSFDT** 1504	**RMET** 32.7 (13.3) **QSFDT** 33.5 (13.7)	**RMET** 66% **QSFDT** 69%	No covariates included in reported outcomes	TestMyBrain Childhood Experiences Questionnaire 25 Item questionnaire adapted from the Conflicts Tactic Scale, the Composite International Diagnostic Interview and Adverse Childhood Experiences (ACE) Scale	Parental maltreatment, parental maladjustment, neglect, sexual abuse/institutional care	RMET (Baron-Cohen et al., 2001), Queen Square Face Discrimination Test (Garrido et al., 2009; Germine & Hooker, 2011)	Theory of Mind, Emotion recognition – Identifying the correct emotion	**RMET total x** Parental Maltreatment: (β = −0.061, *p* = 0.004), Parental Maladjustment: (β = −0.076, *p* = 0.000), Neglect: (β = −0.019, *p* = 0.362), Sexual Abuse/Institutionalisation: (β = −0.045, *p* = 0.031) **QSFDT Test (Emotion Identification) x** Parental Maltreatment: (β = −0.048, *p* = 0.049), Parental Maladjustment: (β = 0.011, *p* = 0.670), Neglect: (β = 0.005, *p* = 0.832), Sexual Abuse/Institutionalisation: (β = −0.037, *p* = 0.127)	Retrospective
**TestMyBrain** Peterson et al., (2022)	Native English speakers only recruited online	2200	Young adults 41.8% aged 18–25	66.3%	Sex (Male/Female), age (Years), ethnicity (Hispanic, White, Black, Other), educational attainment, parents educational attainment, relative SES	TestMyBrain Childhood Experiences Questionnaire 25 Item questionnaire adapted from the Conflicts Tactic Scale, the Composite International Diagnostic Interview and Adverse Childhood Experiences (ACE) Scale	Childhood maltreatment and interpersonal loss	REMT—36 pictures (Baron-Cohen et al., 2001)	Theory of mind	RMET x: Any trauma (β = −0.34 [−0.84, 0.17], *p* = 0.189), Maltreatment (β = −0.21 [−0.66, 0.25], *p* = 0.369), Interpersonal loss (β = −0.25 [−0.61, 0.10], *p* = 0.170), Physical abuse (β = −0.06 [−0.42, 0.30], *p* = 0.740), Sexual abuse (β = −0.31 [−0.75, 0.14], *p* = 0.175)	Retrospective
Turner et al., (2022)	Recruited through social media and Facebook advertisements	197	37.5 (13.7)	57%	None included in correlation of interest	CTQ-SF—28 item questionnaire (Bernstein et al., 2013)	Physical abuse, physical neglect, emotional abuse, emotional neglect, sexual abuse and aggregate childhood trauma score	Computerised Director Task (Dumontheil et al., 2010)	Theory of Mind	Unstandardised beta regression coefficients CTQ total x TOM Performance (β = −0.0028, *p* = 0.0022)	Retrospective
Vaskinn et al., (2021)	Healthy controls randomly selected from the official population registries in Oslo, Norway	70	29.4 (7.7)	40%	None stated	CTQ—28 item questionnaire (Bernstein et al., 2003)	Physical abuse, physical neglect, emotional abuse, emotional neglect, sexual abuse and aggregate childhood trauma score	Movie for the Assessment of Social Cognition (MASC) Norwegian version (Dziobek et al., 2006)	Theory of Mind	MASC total x: CTQ total: (*r*_*s*_ = −0.072,p = 0.562) PA: (*r*_*s*_ = 0.118, *p* = 0.334), SA: (*r*_*s*_ = 0.088, *p* = 0.471), EA: (*r*_*s*_ = 0.041, *p* = 0.739), EN: (*r*_*s*_ = −0.084, *p* = 0.495), PN: (*r*_*s*_ = 0.000, *p* = 0.9999)	Retrospective
Young & Widom, (2014)	Abused/neglected children from juvenile and adult criminal arrest records in Midwestern metropolitan area of the USA	547	47.1 (3.45)	57.8%	Age, Sex, Race and SES	ELA was measured through examination of criminal and court records	Physical abuse, physical neglect, sexual abuse and neglect	International Affective Picture System (IAPS; Lang, Bradley, & Cuthbert, 2005)	Emotion recognition – Correctly identifying emotional valence	Overall picture recognition accuracy x: Abuse/neglect: (β = −0.11, *p* < 0.01, SE = 0.04, n = 547), Physical abuse: (β = −0.08, *p* > 0.05, SE = 0.06, n = 294), Sexual abuse: (β = −0.11, *p* > 0.05, SE = 0.06, n = 301)	Retrospective. ELA was measured through examination of criminal and court records
Zhao & Wu, (2022)	Healthy adults recruited through online advertising from six Chinese universities	173	22.5 (2.2)	55%	Age, sex, education, marital status, recent stressful life events, depression and anxiety levels, personality, cognitive reappraisal and expression inhibition strategy	CTQ—28 item questionnaire (Bernstein et al., 2003)	Physical abuse, physical neglect, emotional abuse, emotional neglect and sexual abuse and aggregate childhood trauma score	International Affective Picture System (IAPS; lang et al., 2008) adapted for a Chinese population (Liu et al., 2009)	Emotion perception – Identifying emotional dominance valence and arousal	CTQ total x IAPS Emotion Score: (r = 0.595, *p* < 0.001), Positive Valence: (β = 0.497, *p* < 0.001), Negative Valence: (β = −0.590, *p* < 0.001), Positive Dominance: (β = 0.424, *p* < 0.001), Negative Dominance: (β = −0.541, *p* < 0.001), Neutral Arousal: (β = −0.434, *p* < 0.001)	Retrospective

**Sample**: CHR Clinically High Risk of neuropsychiatric diagnosis, CHR-NT Clinically High Risk of neuropsychiatric diagnosis- Not Transitioned to a neuropsychiatric diagnosis, SHIP Study of Health in Pomerania, SHIP-LEGENDE Study of Health in Pomerania-Life Events and Gene–Environment Interaction in Depression, SHIP-TREND Study of Health in Pomerania, TREND cohort study. **Covariates:** IQ Intelligence Quotient, MDD Major Depressive Disorder, SES Socioeconomic status. **Adversity Measure:** ACE Adverse Childhood Experience, CECA-Q Childhood Experience of Care and Abuse questionnaire, CTQ Childhood Trauma Questionnaire, CTQ-SF Childhood Trauma Questionnaire—Short Form, ELA Early Life Adversity. **Social Cognition Measures:** CANTAB Cambridge Automated Neuropsychological Test Battery, DERS Difficulties in Emotion Regulation Scale, DFAR Degraded Facial Affect Recognition task, DTQ Disclosure of Trauma Questionnaire, ERT Emotion Recognition Task, HAB Hostile Attribution Biases (Subscale of SIP-AEQ), IAPS International Affective Picture System, IFEEL The Infant Facial Expressions of Emotion from Looking At Pictures, IRI Interpersonal Reactivity Index, MASC Movie for the Assessment of Social Cognition, NER Negative Emotional Responses (Subscale of SIP-AEQ), QSFDT Test Queen Square Face Discrimination Test, RMET Reading the Mind in the Eyes Task, SAQ Social Acknowledgement Questionnaire, SIP-AEQ Social Information Processing-Attribution and Emotional Response Questionnaire, TAS-20 The Toronto Alexithymia Scale. **Social Cognition Domains Measured:** SIP Social Information Processing. **Associations/Correlations:** ER Emotion Recognition, EA Emotional Abuse, EN Emotional Neglect, PA Physical Abuse, PN Physical Neglect, SA Sexual Abuse, B Unstandardised Regression Coefficient, β Beta Regression Coefficient, p P value, r Correlation Coefficient, r_s_ Spearman’s Rho, SD Standard Deviation

### Data Synthesis

The statistical software Jamovi (V2.3.21, https://www.jamovi.org/) was used to analyse data for the meta-analysis. Pearson's correlation coefficient (r) quantifying the association between ELA and social cognition measures was used as the effect size of interest (Pearson, 1909). Any studies reporting alternative Beta coefficients were transformed to correlation coefficients using Peterson & Brown’s formula (r = β + 0.05λ; (Peterson & Brown, 2005)). Spearman’s Rho was equated as a non-parametric equivalent of Pearsons R. Unadjusted estimates were prioritised due to variability in how estimates are adjusted with covariates across studies.

In alignment with previous evidence synthesis work, an overarching measure of social cognition was not pursued (Johnson et al., 2021). This was intended to limit interstudy heterogeneity and ensure that frequently studied subdomains of social cognition were not overly influential in one aggregate measure. Primary analysis consisted of performing a meta-analysis for each domain of social cognition where relevant literature was present. These subdomains included alexithymia, emotion recognition and theory of mind representing different spheres of social cognition.

Fisher’s r-to-z transformed correlation coefficient was the meta-analytic approach chosen (Borenstein et al., 2021; Fisher, 1915; Meng et al., 1992). This approach transforms the sampling distribution so that it becomes normally distributed. An inverse variance weighted random effects model was fitted to the data to provide a mean effect size estimate and account for heterogeneity in methodological approaches and study designs (Borenstein et al., 2021). Integrating inverse variance ensured that studies with larger sample sizes were given more weight. The significance threshold was set at α = 0.05 for significance testing. Effect sizes were reported as Fisher’s r-to-z transformed correlation coefficient (*Z*_*r*_). Forest plots were used to visually represent the results of the meta-analysis, with 95% confidence intervals and the mean-weighted effect estimate also reported. The effect size was considered small, medium and large at integer values of 0.1, 0.3 and 0.5 respectively (Cohen, 1992).

Three studies were required for subgroup analysis, while 10 studies were required for meta-regression analysis. A detailed list of pre-planned subgroup and sensitivity analysis can be found in Supplementary Table 6. The analysis of subgroups was intended to explore potential contributors to inter-study heterogeneity such as age and method used to measure ELA. Sensitivity analysis was intended to explore the impact of removing studies from the analysis that were deemed to be overly influential or outliers upon analysis.

In the occurrence of two reports being published from one study, both reports were included to ensure all relevant information pertaining to a study population was captured. For example, the iRELATE and TestMyBrain publications were treated as one data point.

Statistical heterogeneity was measured using the Q-test for heterogeneity (Cochran, 1954), Hedges tau^2^ estimator of heterogeneity (Hedges & Olkin, 2014) and the I^2^ test statistic (Higgins & Thompson, 2002). The Q-test for heterogeneity calculates the weighted sum of squared differences between individual studies and the pooled effect, with an alpha risk of 0.05 set for significance testing. Hedges tau^2^ estimator of heterogeneity measures between study variance, while the I^2^ test statistic estimates the proportion of variation that is due to actual differences between the included studies and not due to random chance. Studentised residuals and examination of Cook’s distances (Cook, 1977) were used to examine whether studies may be outliers or disproportionately influential given the chosen model. The model fit was analysed using the log-likelihood goodness of fit.

### Assessment of Bias

Publication bias for the meta-analysis was assessed using Rosenthal’s Fail-Safe N calculation (Rosenthal, 1979), Begg and Mazumdar rank correlation (Begg & Mazumdar, 1994), Trim and Fill number of studies (Duval & Tweedie, 2000) and Egger’s Regression (Egger et al., 1997) as calculated using the Jamovi software. An alpha risk of 0.05 was chosen for significance testing. It is recommended that at least 10 studies are present to have sufficient power to detect publication bias as measured using funnel plot asymmetry (Higgins & Green, 2008).

## Results

### Search and Screening Outcomes

**Fig. 1:**
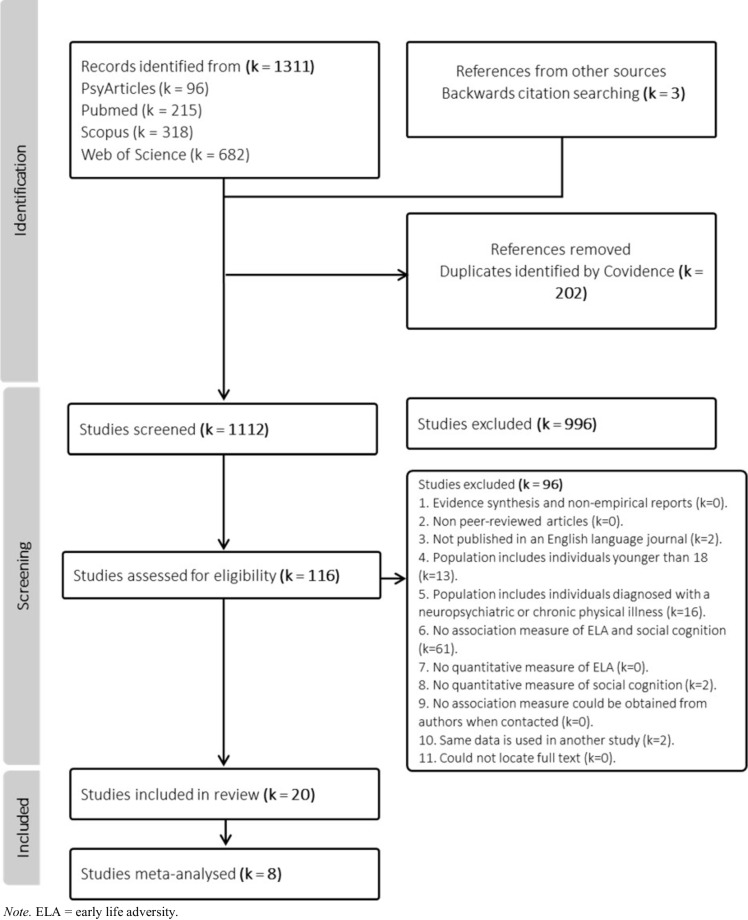
PRISMA flow chart outlining the study selection process

Database searches uncovered 1,314 articles, 202 being duplicates that were removed. Three additional articles were identified through backwards citation searching. 1,112 studies were screened for inclusion at the title/abstract level. 116 articles were assessed for inclusion at the full text level, with 20 studies representing 18 study populations meeting the criteria for inclusion (Fig. 1). Two authors were contacted for additional information. A response was not obtained from one author, with the remaining author providing additional relevant information that resulted in the study being excluded. For an overview of the included studies please see Table 1.

### Study Characteristics

A diverse array of countries were represented in the 18 study populations including; the USA (k = 3), Germany (k = 3), Canada (k = 2), China (k = 2), the United Kingdom (k = 2), Korea (k = 1), Ireland (k = 1), Norway (k = 1) Poland (k = 1), Switzerland (k = 1) and one online study (k = 1) representing multiple international locations.

**Fig. 2:**
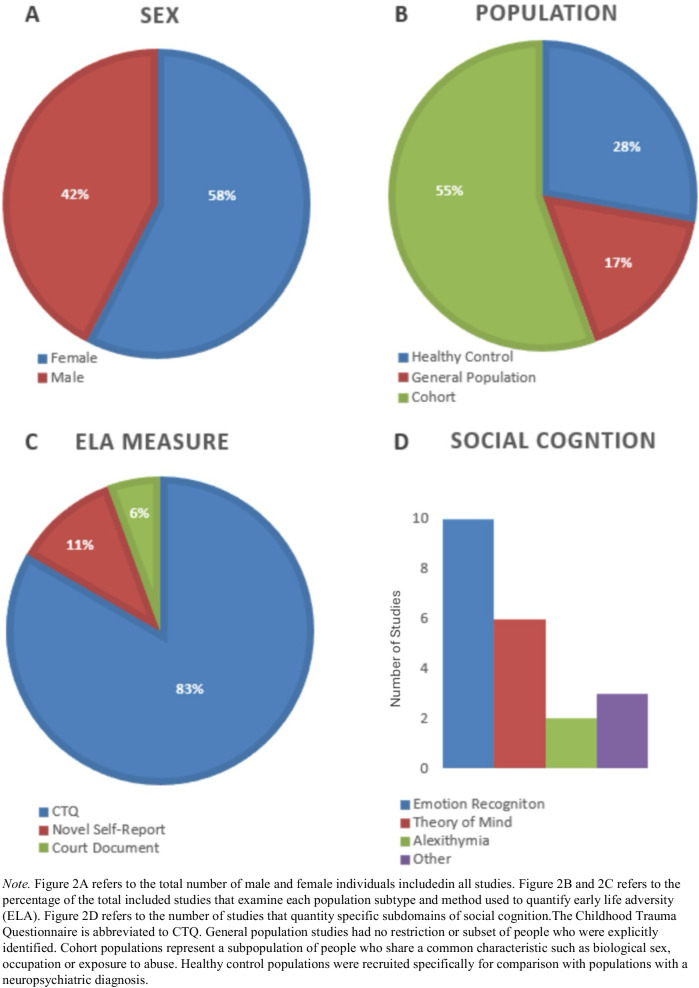
Summary of included studies

Of the 18 study populations included, three studies examined female populations exclusively with the remaining 15 studies examining mixed male and female populations. In total 15,829 participants were included in this systematic review with 58% of participants being female (Fig. 2A).

From the total 18 studies included, 28% (k = 5) of study populations were healthy control groups that were recruited for the study of neuropsychiatric conditions. 17% (k = 3) were general adult populations, with 55% (k = 10) of studies recruiting cohort populations (Fig. 2B). These cohort studies included female only populations (k = 3), studies recruited exclusively from university populations (k = 2) and studies that examined populations where all participants were selected based on their exposure to abuse (k = 2). Studies that examined a cohort of twins (k = 1), a mother and child cohort (k = 1) and individuals specifically not taking psychiatric medication (k = 1) were also included (Fig. 2B).

All 18 studies retrospectively examined early life adversity with 17 studies using self-report measures. 83% (k = 15) of studies used the Childhood Trauma Questionnaire (CTQ) to retrospectively measure ELA. The English (k = 5), German (k = 4), Chinese (k = 2), French (k = 1), Korean (k = 1) Norwegian (l = 1) and Polish (k = 1) versions of the CTQ were used. 11% (k = 2) of studies used novel scales to quantify ELA, with 6% of studies (k = 1), using court documents to objectively quantify ELA (Fig. 2C).

In total 10 studies examined emotion recognition as a domain of social cognition. Six studies examined theory of mind, two studies examined alexithymia and three studies examined alternatives spheres of social cognition (Fig. 2D).

56% of studies (k = 10) reported Pearson’s correlations, 22% (k = 4) reported standardised beta coefficients and 11% (k = 2) reported an unstandardised beta regression coefficient. 6% (k = 1) of studies reported an unadjusted regression coefficient and Spearman’s rho correlation coefficient (k = 1) respectively. Of these association measures, nine were adjusted for covariates while nine studies did not include covariates in their association measures.

### Meta Analysis: ELA and Emotion Recognition Total

**Fig. 3:**
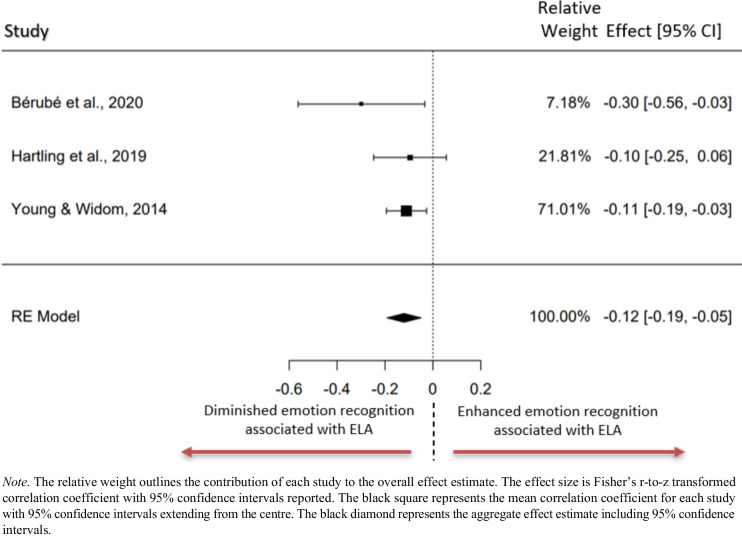
Forest plot representing the meta-analysis examining the relationship between emotion recognition accuracy and ELA total exposure

Three studies were included in the meta-analysis examining the association between ELA and emotion recognition (Fig 3). All three studies measured the association between cumulative ELA exposure and emotion recognition accuracy total score. The observed Fisher r-to-z transformed correlation coefficients ranged from −0.299 to −0.095 with all reported estimates being negative. The estimated average Fisher r-to-z transformed correlation coefficient based on the random-effects model was *z*_*r*_ = −0.121, 95% CI[−0.192, −0.050]. The average outcome differed significantly from zero (*z* = −3.339, *p* < 0.001). A 95% prediction interval for the true outcomes was estimated to reside between −0.192 to −0.050. Examination of the studentised residuals revealed that no studies had a value larger than ± 2.394, which indicates that no studies were outliers in the context of this model. Examination of the Cook’s distances revealed that no study could be considered overly influential.

### Meta-analysis: Sexual Abuse and Emotion Recognition Total

**Fig. 4:**
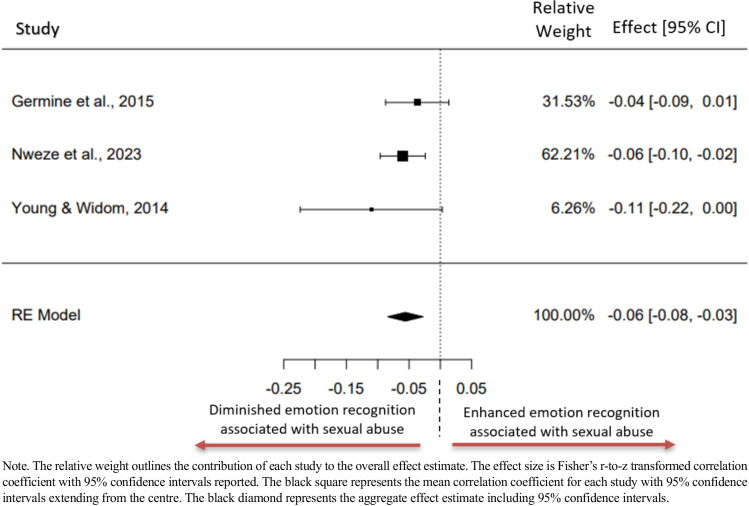
Forest plot representing the meta-analysis examining the relationship between emotion recognition accuracy and sexual abuse exposure

There was enough relevant data to explore sexual abuse as a subdomain of ELA and examine its association with emotion recognition accuracy (Fig 4). The three studies included in this analysis all reported a negative association between sexual abuse and emotion recognition. The subgroup of relevance from the Germine and colleagues study consisted of adults that self-reported experiencing sexual abuse or institutional care or both, meaning their construct did not solely reflect experiences of sexual abuse (Germine et al., 2015).

Using the random effects model, the estimated average Fisher r-to-z transformed correlation coefficient was: *z*_*r*_ = −0.056, 95%CI[−0.084, −0.028]. The average outcome differed significantly from zero (*z* = −3.861*, p* < 0.001). Examination of the studentised residuals revealed that no study had a value larger than ± 2.394, indicating that no studies could be considered an outlier in the context of this model. According to the Cook's distances, none of the studies could be considered to be overly influential.

### Meta-analysis: ELA and Theory of Mind

**Fig. 5:**
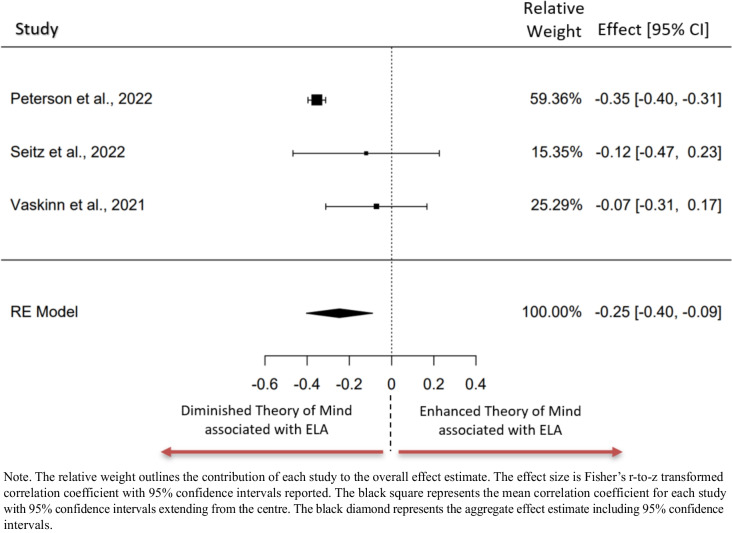
Forest plot representing the meta-analysis examining the relationship between Theory of Mind and ELA total exposure

Three studies were included in the analysis examining the relationship between ELA total exposure and theory of mind (Fig 5). The observed Fisher r-to-z transformed correlation coefficients ranged from −0.354 to −0.072, with all estimates being negative. The estimated average Fisher r-to-z transformed correlation coefficient based on the random-effects model was *z*_*r*_ = −0.247, 95%CI[−0.403 to −0.090]. The average outcome differed significantly from zero (*z* = −3.092*, p* = 0.002). A 95% prediction interval for the true outcomes is given by −0.500 to 0.006. Hence, although the average outcome is estimated to be negative, in some studies the true outcome may in fact be positive. An examination of the studentised residuals revealed that one study (Peterson et al., 2022) had a value larger than ± 2.394 and may be a potential outlier in the context of this model. No study was considered overly influential upon examination of Cook’s distances.

### Publication Bias

No evidence of publication bias was present in the meta-analyses (see Table 2). Rosenthal’s fail-safe N calculation reached the significance threshold for all three meta-analyses conducted indicating that the observed effect size is unlikely to be due to publication bias. It is recommended that 10 studies are required to have sufficient power to detect funnel plot asymmetry and publication bias (Higgins & Green, 2008). The minimum study requirement number was not met in this case, highlighting the need to interpret these results with extreme caution.

**Table 2:** Assessment of Heterogeneity and Publication Bias

ELA Exposure	Social Cognition Measure	Publication Bias Assessment	Heterogeneity Assessment
ELA total	Emotion Recognition total	Fail-Safe N = 11*, p* < 0.001	Q-test for heterogeneity Q(2) = 1.905*, p* = 0.386 tau^2^ = 0.000 I^2^ = 0.018%
Sexual Abuse	Emotion Recognition total	Fail-Safe N = 14*, p* < 0.001	Q-test for heterogeneity: Q(2) = 1.4734*, p* = 0.479 tau^2^ = 0.000 I^2^ = 0.000%
ELA total	Theory of Mind total	Fail-Safe N = 116*, p* < 0.001	Q-test for heterogeneity: (Q(2) = 6.769*, p* = 0.034 tau^2^ = 0.010 I^2^ = 49.599%

### Subgroup and Sensitivity Analysis

Pre-planned subgroup and sensitivity analysis detailed in Supplementary Table 6 was precluded due to the limited availability of studies.

## Discussion

To our knowledge, this is the first systematic review and meta-analysis to examine the relationship between early life adversity and social cognition in the general adult population. This systematic review and meta-analysis found that ELA exposure was associated with diminished emotion recognition and theory of mind in the general adult population. Sexual abuse as a subdomain of ELA was also found to be significantly associated with diminished emotion recognition ability. In congruence with previous research in populations with a neuropsychiatric diagnosis, ELA was found to be associated with diminished social cognitive ability (Rokita et al., 2018). Novel to this evidence synthesis article is the presence of this association in the general population.

Caution is necessary when interpreting these results due to the limited number of studies included in the meta-analysis. Despite 20 studies being suitable for inclusion, only three studies were included per meta-analysis due to heterogeneity in study design. More research is required to appropriately substantiate these findings and increase the statistical power to detect true associations. With 12 of the 20 included studies published since 2020, an emerging interest in this topic area is clear.

Research examining ELA’s relationship with social cognition has predominantly focused on populations with a neuropsychiatric diagnosis. A negative association between ELA and social cognition has been reported trans-diagnostically in these populations, however there is little understanding to the causative nature of social cognition deficits (Catalana et al., 2020; Rokita et al., 2018). Given the importance of social cognition for social functioning, independent living and work functioning, a crucial next research step will be to determine if ELA is a modifiable antecedent of social cognitive deficits (Fett et al., 2011). Undoubtably inherent social cognitive deficits can exist within neurodivergent populations and neurological conditions (Henry et al., 2016; Pagni et al., 2020). The occurrence of ELA during crucial developmental stages has led to research examining the potential for altered neurodevelopmental trajectories with included study conducted by Nweze et al., 2023 also identifying timing of exposure to ELA as being crucial (Malave et al., 2022; Pollard et al., 2022). Discerning if social cognitive deficits observed in the general population associated with ELA are inherent, are neurodevelopmentally influenced or occur as a result of adaptive coping strategies is of crucial importance and has the potential to guide strategies for prevention and therapeutic intervention. Social cognition deficits have already been explored as a therapeutic target for early psychosis in clinical populations (Yamada et al., 2019).

From a neurobiological perspective, inflammation is being explored as a potential mechanism linking ELA and social cognitive deficits. Experimentally induced low-grade inflammation through vaccination or endotoxin exposure has been shown to have a temporal and reversible deleterious effect on social cognition in healthy population studies (Balter et al., 2018; Kullmann et al., 2014; Moieni et al., 2015). Furthermore, in individuals diagnosed with schizophrenia, inflammation was shown to partially mediate the relationship between ELA and emotion recognition deficits, and between ELA and default mode network connectivity during a theory of mind task (King et al., 2021, 2022). Research exploring inflammation as a mediator of the relationship between ELA and social cognition in the general population would help to develop understanding of underlying causal mechanisms. A phenotype of inflammation induced social cognitive underperformance associated with ELA merits further exploration in the general population.

Examining the association between ELA and social cognition in non-clinical populations allows determinants of social cognition to be explored in the absence of psychiatric classifications. Psychiatric diagnosis has been heavily criticised for the broad and heterogenous grouping of individuals, the undetermined aetiopathogenesis of psychiatric groups, and the failure of these categories to locate causes of distress (Allsopp et al., 2019; Johnstone & Boyle, 2018; Wardenaar & de Jonge, 2013). An ‘overreliance on diagnosis specific research’ has been discussed with longitudinal research identifying high comorbidity and dynamic presentation of symptoms that encompass a multitude of diagnostic categories over a person’s life course (Caspi et al., 2020; Plana-Ripoll et al., 2019). Both the British Psychological Society’s (BPS) Division of Clinical Psychology and a United Nations report have called for a paradigm shift away from the psychiatric disease model for conceptualising distress, with the UN report emphasising the need to further understand the social determinants of health (DCP, 2013; Johnstone & Boyle, 2018; Office of the High Commisioner for Human Rights, 2017).

The Childhood Trauma Questionnaire (CTQ) was the predominant measure used to quantify ELA in this systematic review and meta-analysis (Bernstein et al., 1994). The CTQ incorporates both the severity and frequency of each traumatic experience which prohibits us from discerning the individual contribution of severity and frequency of ELA to social cognition.

### Limitations and Future Research

Inter-study heterogeneity was a limitation of this review article. A substantial proportion of variance between studies in the meta-analysis examining the association between ELA and theory of mind was not explained by random chance but was due to actual differences in the included studies designs and populations. The limited number of included studies prevented the exploration of heterogeneity through subgroup and sensitivity analysis. Although none of the included studies reported that covariates influenced social cognitive scores, variables including age (Grainger et al., 2023), sex (Paletta et al., 2023) and socioeconomic status (Migeot et al., 2022) have been shown to influence social cognition and may have contributed to interstudy heterogeneity.

Secondly, our study is more representative of significant adversities that an individual may have experienced. While our operational definition of ELA was broad to represent the multitude of adversities that people experience, 83% of studies used the CTQ to retrospectively quantify five domains of ELA. This review has not captured harder to recognise and more insidious forms of ELA including financial difficulties, legal concerns, bullying, the breakdown of significant relationships or living within an oppressive system (Dhawan & Sinha, 2022). Further research specifically examining these subtypes of ELA is necessary to understand their association with social cognition.

Exploring mediators of the relationship between ELA and social cognition, such as psycho-social mechanisms (e.g. coping), neurobiological mechanisms (e.g. inflammation) and exploring the impact of timing and type of adversity experienced on social cognition warrants further investigation. There is evidence that certain developmental periods and adversity types propound a disproportionate influence on adult mental health indices (Maercker et al., 2004; Schalinski et al., 2016). Exploring how the timing and type of adversity may differentially influence social cognition is crucial to understanding how ELA may influence neurodevelopmental trajectories. One of our included studies reported that the timing of ELA exposure explained more of the association between ELA and social cognition than the number of adversities that a person experiences, necessitating the need for exploration of developmentally sensitive periods (Nweze et al., 2023).

Finally, longitudinal prospective research will be crucial in delineating causal mechanisms underlying ELA’s association with social cognition. This research design would allow for altered neurodevelopment and inflammation induced theories of ELA’s association with social cognitive deficits to be explored. The present cross-sectional design of included studies prevents any causal associations being made.

## Conclusion

ELA is associated with diminished theory of mind and emotion recognition ability in the general population and specifically, experiences of sexual abuse were associated with diminished emotion recognition. Correlational findings on the association between retrospective accounts of ELA and social cognitive deficits during adulthood suggest a continuing impact of trauma in later life. Due to the limited number of studies and varying levels of between-study heterogeneity, it is necessary to interpret these results with caution. Longitudinal research is best situated to further explore causative and mediating factors underlying the ELA-social cognition association.

## Supplementary Information

Below is the link to the electronic supplementary material.

Supplementary file1 (DOCX 65 KB)

## Data Availability

Analysed datasets from the current study are available at https://osf.io/6y7x5/?view_only=8eb9088d51c54648be6f74bb17d80e05. Additional datasets generated during the study are available from the corresponding author upon reasonable request.
