# Supplementary material for: ‘Early Life Adversity and Social Cognition in the General Adult Population: A Systematic Review and Meta-Analysis’
Source: J Child Adolesc Trauma. 2025 Jun 24;18(4):925–45. doi: 10.1007/s40653-025-00724-y (PMC12831749; doi:10.1007/s40653-025-00724-y)
Supplement: Supplementary file 1 — Supplementary file1 (DOCX 65 KB) [file 40653_2025_724_MOESM1_ESM.docx]

**Early life adversity and social cognition in the general adult population: A systematic review and meta-analysis**

**Supplementary Materials**

**Supplementary Table 1:** PRISMA Checklist

**Supplementary Table 2:** Database Search Strategy

**Supplementary Table 3:** Inclusion and Exclusion Criteria for Article Selection

**Supplementary Table 4:** Population, Exposure, Comparator, and Outcome (PECO) Details

**Supplementary Table 5:** Exclusion Hierarchy

**Supplementary Table 6:** Pre-planned Subgroup and Sensitivity Analysis

**Supplementary Table 7:** Studies Included in Each Section of the Systematic Review and Meta-analysis

**Supplement 1:** Deviations from Preregistration

| **Section and Topic**  **Supplementary Table 1** – PRISMA Checklist (Page et al., 2021) | **Item #** | **Checklist item** | **Location (Section)** |
| --- | --- | --- | --- |
| **TITLE** | | |  |
| Title | 1 | Identify the report as a systematic review. | TITLE |
| **ABSTRACT** | | |  |
| Abstract | 2 | See the PRISMA 2020 for Abstracts checklist. | ABS |
| **INTRODUCTION** | | |  |
| Rationale | 3 | Describe the rationale for the review in the context of existing knowledge. | INTRO |
| Objectives | 4 | Provide an explicit statement of the objective(s) or question(s) the review addresses. | INTRO |
| **METHODS** | | |  |
| Eligibility criteria | 5 | Specify the inclusion and exclusion criteria for the review and how studies were grouped for the syntheses. | METHODS |
| Information sources | 6 | Specify all databases, registers, websites, organisations, reference lists and other sources searched or consulted to identify studies. Specify the date when each source was last searched or consulted. | METHODS |
| Search strategy | 7 | Present the full search strategies for all databases, registers and websites, including any filters and limits used. | METHODS |
| Selection process | 8 | Specify the methods used to decide whether a study met the inclusion criteria of the review, including how many reviewers screened each record and each report retrieved, whether they worked independently, and if applicable, details of automation tools used in the process. | METHODS |
| Data collection process | 9 | Specify the methods used to collect data from reports, including how many reviewers collected data from each report, whether they worked independently, any processes for obtaining or confirming data from study investigators, and if applicable, details of automation tools used in the process. | METHODS |
| Data items | 10a | List and define all outcomes for which data were sought. Specify whether all results that were compatible with each outcome domain in each study were sought (e.g. for all measures, time points, analyses), and if not, the methods used to decide which results to collect. | METHODS |
|  | 10b | List and define all other variables for which data were sought (e.g. participant and intervention characteristics, funding sources). Describe any assumptions made about any missing or unclear information. | METHODS |
| Study risk of bias assessment | 11 | Specify the methods used to assess risk of bias in the included studies, including details of the tool(s) used, how many reviewers assessed each study and whether they worked independently, and if applicable, details of automation tools used in the process. | METHODS |
| Effect measures | 12 | Specify for each outcome the effect measure(s) (e.g. risk ratio, mean difference) used in the synthesis or presentation of results. | METHODS |
| Synthesis methods | 13a | Describe the processes used to decide which studies were eligible for each synthesis (e.g. tabulating the study intervention characteristics and comparing against the planned groups for each synthesis (item #5)). | METHODS |
|  | 13b | Describe any methods required to prepare the data for presentation or synthesis, such as handling of missing summary statistics, or data conversions. | METHODS |
|  | 13c | Describe any methods used to tabulate or visually display results of individual studies and syntheses. | METHODS |
|  | 13d | Describe any methods used to synthesize results and provide a rationale for the choice(s). If meta-analysis was performed, describe the model(s), method(s) to identify the presence and extent of statistical heterogeneity, and software package(s) used. | METHODS |
|  | 13e | Describe any methods used to explore possible causes of heterogeneity among study results (e.g. subgroup analysis, meta-regression). | METHODS |
|  | 13f | Describe any sensitivity analyses conducted to assess robustness of the synthesized results. | METHODS |
| Reporting bias assessment | 14 | Describe any methods used to assess risk of bias due to missing results in a synthesis (arising from reporting biases). | METHODS |
| Certainty assessment | 15 | Describe any methods used to assess certainty (or confidence) in the body of evidence for an outcome. | METHODS |
| **RESULTS** | | |  |
| .Study selection | 16a | Describe the results of the search and selection process, from the number of records identified in the search to the number of studies included in the review, ideally using a flow diagram. | RESULTS |
|  | 16b | Cite studies that might appear to meet the inclusion criteria, but which were excluded, and explain why they were excluded. | RESULTS |
| Study characteristics | 17 | Cite each included study and present its characteristics. | RESULTS |
| Risk of bias in studies | 18 | Present assessments of risk of bias for each included study. | RESULTS |
| Results of individual studies | 19 | For all outcomes, present, for each study: (a) summary statistics for each group (where appropriate) and (b) an effect estimate and its precision (e.g. confidence/credible interval), ideally using structured tables or plots. | RESULTS |
| Results of syntheses | 20a | For each synthesis, briefly summarise the characteristics and risk of bias among contributing studies. | RESULTS |
|  | 20b | Present results of all statistical syntheses conducted. If meta-analysis was done, present for each the summary estimate and its precision (e.g. confidence/credible interval) and measures of statistical heterogeneity. If comparing groups, describe the direction of the effect. | RESULTS |
|  | 20c | Present results of all investigations of possible causes of heterogeneity among study results. | RESULTS |
|  | 20d | Present results of all sensitivity analyses conducted to assess the robustness of the synthesized results. | RESULTS |
| Reporting biases | 21 | Present assessments of risk of bias due to missing results (arising from reporting biases) for each synthesis assessed. | RESULTS |
| Certainty of evidence | 22 | Present assessments of certainty (or confidence) in the body of evidence for each outcome assessed. | RESULTS |
| **DISCUSSION** | | |  |
| Discussion | 23a | Provide a general interpretation of the results in the context of other evidence. | DISCUSS |
|  | 23b | Discuss any limitations of the evidence included in the review. | DISCUSS |
|  | 23c | Discuss any limitations of the review processes used. | DISCUSS |
|  | 23d | Discuss implications of the results for practice, policy, and future research. | DISCUSS |
| **OTHER INFORMATION** | | |  |
| Registration and protocol | 24a | Provide registration information for the review, including register name and registration number, or state that the review was not registered. | METHODS |
|  | 24b | Indicate where the review protocol can be accessed, or state that a protocol was not prepared. | METHODS |
|  | 24c | Describe and explain any amendments to information provided at registration or in the protocol. | SUPPLEM |
| Support | 25 | Describe sources of financial or non-financial support for the review, and the role of the funders or sponsors in the review. | DISCLOSE |
| Competing interests | 26 | Declare any competing interests of review authors. | DISCLOSE |
| Availability of data, code and other materials | 27 | Report which of the following are publicly available and where they can be found: template data collection forms; data extracted from included studies; data used for all analyses; analytic code; any other materials used in the review. | DISCLOSE |

**Supplementary Table 2.** Search strategy terms for PubMed, PsycArticles Scopus and the Web of Science (WOS) databases.

|  | Study Population | Exposure: Adversity | Outcome: Social Cognition |
| --- | --- | --- | --- |
| **PsycArticles** | Healthy OR Control OR Controls OR Adult OR General Population | Childhood Trauma OR Maltreatment OR Adverse OR Adversity OR Abandonment OR Neglect OR Stressful OR Life Event OR Early Life | Social Cognition OR Cognition OR Social Affect OR Theory of Mind OR Emotion Perception OR Affect Perception OR Emotion Recognition OR Affect Recognition OR Emotion Processing OR Affect Processing OR Attributional style |
|  | **Exact Advanced Search:**  ((abstract: childhood trauma OR abstract: maltreatment OR abstract: adverse OR abstract: adversity OR abstract: Abandonment OR abstract: neglect OR abstract: Stressful OR abstract: Life Event OR abstract: Early life) AND (abstract: social cognition OR abstract: Cognition OR abstract: social affect OR abstract: theory of mind OR abstract: emotion perception OR abstract: affect perception OR abstract: emotion recognition OR abstract: affect recognition OR abstract: emotion processing OR abstract: emotion processing OR abstract: attributional style) AND (abstract: healthy OR abstract: control OR abstract: controls OR abstract: adult OR abstract: general population)) | | |
| **PubMed** | Healthy OR Control OR Controls OR Adult OR General Population | Childhood Trauma OR Maltreatment OR Adverse OR Adversity OR Abandonment OR Neglect OR Stressful OR Life Event OR Early Life | Social Cognition OR Cognition OR Social Affect OR Theory of Mind OR Emotion Perception OR Affect Perception OR Emotion Recognition OR Affect Recognition OR Emotion Processing OR Affect Processing OR Attributional style |
|  | **Exact Advanced Search:**  ((Childhood Trauma[Title/Abstract] OR Maltreatment[Title/Abstract] OR Childhood Abuse[Title/Abstract] OR Adverse[Title/Abstract] OR Adversity[Title/Abstract] OR Abandonment[Title/Abstract] OR Neglect[Title/Abstract] OR Stressful[Title/Abstract] OR Life Event[Title/Abstract] OR Early Life[Title/Abstract]) AND (Social Cognition[Title/Abstract] OR Social Affect[Title/Abstract] OR Emotion Perception[Title/Abstract] OR Affect Perception[Title/Abstract] OR Emotion Processing[Title/Abstract] OR Affect Processing[Title/Abstract] OR Emotion Recognition[Title/Abstract] OR Affect Recognition[Title/Abstract] OR Attributional style[Title/Abstract] OR Theory of Mind[Title/Abstract]) AND (Healthy[Title/Abstract] OR Control[Title/Abstract] OR Controls[Title/Abstract] OR Adult[Title/Abstract] OR General Population[Title/Abstract]) AND (Association OR Correlation)) | | |
| **Scopus** | Healthy OR Control OR Controls OR Adult OR General Population | Childhood Trauma OR Maltreatment OR Adverse OR Adversity OR Abandonment OR Neglect OR Stressful OR Life Event OR Early Life | Social Cognition OR Social Affect OR Theory of Mind OR Emotion Perception OR Affect Perception OR Emotion Recognition OR Affect Recognition OR Emotion Processing OR Affect Processing OR Attributional style |
|  | **Exact Advanced Search:**  TITLE-ABS-KEY ( ("childhood trauma" OR maltreatment OR "childhood abuse" OR adverse OR adversity OR abandonment OR neglect OR stressful OR "Life Event") AND ( "social cognition" OR “social affect” OR "emotion perception" OR "affect perception" OR "emotion recognition" OR "affect recognition" OR "emotion processing" OR "affect processing" OR "attributional style" OR "Theory of Mind") AND ( healthy OR control OR controls OR adult OR "general population" ) AND (association OR Correlation) ) | | |
| **Web of Science** | Healthy OR Control OR Controls OR Adult OR General Population | Childhood Trauma OR Maltreatment OR Adverse OR Adversity OR Abandonment OR Neglect OR Stressful OR Life Event OR Early Life | Social Cognition OR Social Affect OR Theory of Mind OR Emotion Perception OR Affect Perception OR Emotion Recognition OR Affect Recognition OR Emotion Processing OR Affect Processing OR Attributional style |
|  | **Exact Advanced Search:**  AB =( ( Childhood Trauma OR Maltreatment OR Childhood Abuse OR Adverse OR Adversity OR Abandonment OR Neglect OR Stressful OR Life Event) AND (Social OR Cognition) AND (Social Affect OR Emotion Perception OR Affect perception OR Emotion Recognition OR Affect Recognition OR Emotion Processing OR Affect processing OR Attributional style OR Theory of Mind ) AND (Healthy OR Control OR Controls OR adult OR general population ) AND (Association OR Correlation) ) | | |

**Supplementary Table 3.** Inclusion and Exclusion Criteria for Article Selection

| **Inclusion Criteria** |
| --- |
| 1. Adult sample ≥ 18 years of age at time of participation. 2. Quantitative measurement of exposure to ELA reported. 3. Quantitative measurement of scores in social cognition tasks reported. 4. Quantitative measure of the association between ELA and social cognition test scores reported. If no association measure was reported, studies were included if relevant data was present that would allow for calculation of the effect size. Otherwise, study authors were contacted to request necessary measures. 5. Studies including individuals deemed to be at a higher risk for neuropsychiatric disorder diagnosis were included if no clinical diagnosis was present (e.g first degree relatives of individuals with a neuropsychiatric diagnosis have been considered to be at a higher lifetime risk of receiving a neuropsychiatric diagnosis than the general population). 6. Peer reviewed article. 7. English language article. |
| **Exclusion Criteria:** |
| 1. Non-adult sample at time of participation (<18 years old). 2. Individuals within the population diagnosed with a neuropsychiatric disorder according to the DSM or ICD. 3. Individuals within the population diagnosed with a chronic physical illness including autoimmune disorders, cancer, diabetes, heart disease and neurological disorders. 4. No measurement of ELA reported. 5. No measurement of social cognition reported. 6. No association measure of ELA and social cognition reported or could not be obtained upon request from the author. 7. Qualitative articles. 8. Non-English language articles. 9. Non-peer reviewed articles. |

## **Appendix D**

**Supplementary Table 4.** Population, Exposure, Comparator, and Outcome (PECO) Details

| Population | Studies that consist of human participants aged eighteen years old and older at the time of participation from any geographic location. Studies including individuals deemed to be at a higher risk for neuropsychiatric disorder diagnosis were included if no clinical diagnosis was present (e.g first degree relatives of individuals with a neuropsychiatric diagnosis have been considered to be at a higher lifetime risk of receiving a neuropsychiatric diagnosis than the general population). Studies including individuals diagnosed with a neuropsychiatric disorder according to the DSM or ICD were excluded. Studies including participants with chronic health diagnoses including autoimmune disorders, cancer, diabetes, heart disease and neurological disorders were also excluded. |
| --- | --- |
| Exposure | Early life adversity (ELA) was operationally defined as exposure to severe adverse experiences that deviate from an expected environment prior to reaching eighteen years of age, necessitating significant psychological and biological adaptation (McLaughlin, 2016; McLaughlin, 2018). Various forms of ELA including but not limited to: abuse (physical, emotional and sexual), neglect (physical and emotional), institutionalisation, foster care, food insecurity, bereavement and socioeconomic deprivation experienced between birth and eighteen years old were included. |
| Comparator | Not applicable. |
| Outcome | Our main outcome is to measure the strength and direction of the relationship between scores on early life adversity measures and social cognition tests. Fisher’s r-to-z transformed correlation coefficient was the meta-analytic approach chosen to measure the aggregate effect. An inverse variance weighted random effects model was fitted to the data to provide a mean effect size estimate, with 95% confidence intervals reported. |

**Supplementary Table 5**. Exclusion Hierarchy

| 1. Evidence synthesis articles and non-empirical reports.  2. Non peer-reviewed articles.  3. Article is not published in an English language journal.  4. Population includes individuals younger than 18 years old at the time of participation.  5. Population includes individuals diagnosed with a neuropsychiatric or chronic physical illness at time of participation.  6. No association measure of ELA and social cognition.  7. No quantitative measure of ELA.  8. No quantitative measure of social cognition.  9. No association measure could be obtained from authors when contacted.  10. Same data is used in another study.  11. Could not locate full-text. |
| --- |

**Supplementary Table 6.** Pre-planned Subgroup and Sensitivity Analysis

| **Sensitivity Analysis** |
| --- |
| 1. Removing studies whereby the Beta coefficient has been transformed to Pearson's R.  2. Removing studies deemed to be overly influential or outliers upon examining studentised residuals. |
| **Subgroup Analysis** |
| 1. Measures used for social cognition and early life adversity (validated measures versus novel measures).  2. Population risk of neuropsychiatric diagnosis (high risk versus no reported risk).  3. Age at time of participation in study (young people with a mean age of less than thirty years old versus people older than thirty).  4. Age reported when early life adversity was experienced (children aged twelve years old and younger versus teenagers aged thirteen to eighteen). |

**Supplementary Table 7.** All Studies Included in the Systematic Review and Meta-analysis.

| **Systematic review:** (Bérubé et al., 2020; Chen et al., 2012; Corley et al., 2024; Dayton et al., 2016; English et al., 2018; Germine et al., 2015; Hartling et al., 2019; Kopera et al., 2020; Krammer et al., 2016; Lee et al., 2019; Liu et al., 2023; Nweze et al., 2023; Peterson et al., 2022; Rokita et al., 2021; Seitz et al., 2022; Terock et al., 2020; Turner et al., 2022; Vaskinn et al., 2021; Young & Widom, 2014; Zhao & Wu, 2022). |
| --- |
| **Meta-analysis examining ELA and Theory of Mind:** (Peterson et al., 2022; Seitz et al., 2022; Vaskinn et al., 2021). |
| **Meta-analysis examining ELA and emotion recognition:** (Bérubé et al., 2020; Hartling et al., 2019; Young & Widom, 2014). |
| **Meta-analysis examining sexual abuse and emotion recognition:** (Germine et al., 2015; Nweze et al., 2023; Young & Widom, 2014). |

**Supplement 1:** Deviations from Preregistration

Our preregistered protocol specified the search strategy, statistical analysis and operational definitions used in our study. We deviated from our preregistered protocol in four instances which are detailed below.

Firstly, the final search of the databases was detailed during preregistration as occurring on the 18th of May 2023. Our final search occurred on the 31st of October 2023 to ensure the most relevant and up to date studies were included.

Secondly, during preregistration it was detailed that qualitative information would be extracted independently by both reviewers and quantitative information would be extracted by R1 and verified by R2. In our study both qualitative and quantitative information was extracted by R1 with second person verification performed by R2. This allowed a consistent approach to the extraction of both qualitative and quantitative data.

Thirdly, it was stated during preregistration that two studies were required for subgroup analysis. We altered the minimum study requirement to three studies for subgroup analysis to ensure sufficient statistical power for significance testing was present and to minimise the risk of both type I and type II errors occurring (Cuijpers et al., 2021).

Finally, subgroup analysis of biological sex (male versus female) was not included in our preregistered protocol however, due to the empirical evidence of sex differences in social cognition it was included in our study (Paletta et al., 2023).

**References**

Cuijpers, P., Griffin, J. W., & Furukawa, T. A. (2021). The lack of statistical power of subgroup analyses in meta-analyses: a cautionary note. Epidemiology and Psychiatric Sciences, 30, e78, Article e78. https://doi.org/10.1017/S2045796021000664

Page, M. J., McKenzie, J. E., Bossuyt, P. M., Boutron, I., Hoffmann, T. C., Mulrow, C. D., Shamseer, L., Tetzlaff, J. M., Akl, E. A., Brennan, S. E., Chou, R., Glanville, J., Grimshaw, J. M., Hróbjartsson, A., Lalu, M. M., Li, T., Loder, E. W., Mayo-Wilson, E., McDonald, S., . . . Moher, D. (2021). The PRISMA 2020 statement: an updated guideline for reporting systematic reviews. BMJ, 372, n71. <https://doi.org/10.1136/bmj.n71>

Paletta, P., Bass, N., Aspesi, D., & Choleris, E. (2023). Sex Differences in Social Cognition. Curr Top Behav Neurosci, 62, 207-234. https://doi.org/10.1007/7854_2022_325
